# Glycoblocks: a schematic three-dimensional representation for glycans and their interactions

**DOI:** 10.1107/S2059798316013553

**Published:** 2016-09-29

**Authors:** Stuart McNicholas, Jon Agirre

**Affiliations:** a York Structural Biology Laboratory, Department of Chemistry, The University of York, York YO10 5DD, England

**Keywords:** Glycoblocks, molecular graphics, glycans, interactions, *CCP4mg*, *Privateer*, carbohydrates, three-dimensional representations

## Abstract

The close-range interactions provided by covalently linked glycans are essential for the correct folding of glycoproteins and also play a pivotal role in recognition processes. Being able to visualise protein–glycan and glycan–glycan contacts in a clear way is thus of great importance for the understanding of these biological processes. In structural terms, glycosylation sugars glue the protein together *via* hydrogen bonds, whereas non-covalently bound glycans frequently harness additional stacking interactions. Finding an unobscured molecular view of these multipartite scenarios is usually far from trivial; in addition to the need to show the interacting protein residues, glycans may contain many branched sugars, each composed of more than ten non-H atoms and offering more than three potential bonding partners. With structural glycoscience finally gaining popularity and steadily increasing the deposition rate of three-dimensional structures of glycoproteins, the need for a clear way of depicting these interactions is more pressing than ever. Here a schematic representation, named Glycoblocks, is introduced which combines a simplified bonding-network depiction (covering hydrogen bonds and stacking interactions) with the familiar two-dimensional glycan notation used by the glycobiology community, brought into three dimensions by the CCP4 molecular graphics project (*CCP4mg*).

## Abstract 2

With structural glycoscience finally gaining popularity, the need for a clear way of depicting glycans and their interactions in three dimensions is more pressing than ever. Here the Glycoblocks representation is introduced, which combines a simplified bonding-network depiction with the familiar two-dimensional glycan notation brought into three-dimensions.

## 1. Introduction   

**Figure 1:**
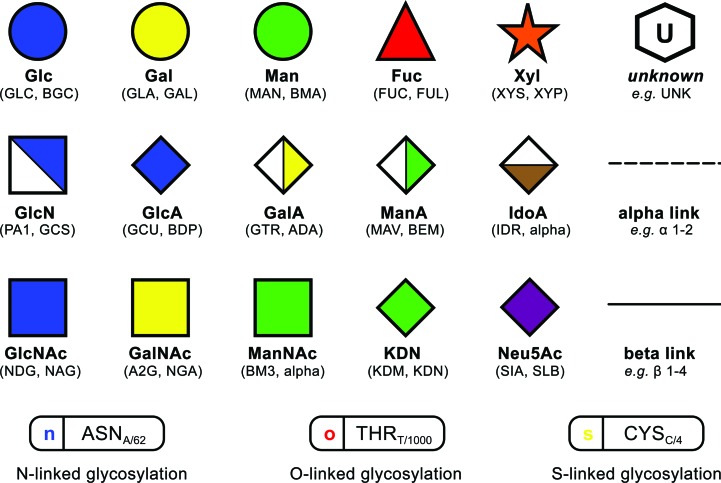
Legend to the two-dimensional representation as drawn by *Privateer*, and its correspondence to three-letter codes from the PDB Chemical Component Dictionary. The current version of *Privateer* adopts all features of the Essentials notation except the bond–angle relation, which will be available in a forthcoming update to the software. Those sugars typically found in both anomeric forms in covalently bound glycans have both three-letter codes assigned to the same shape, *e.g.* GLC (α-d-glucopyranose) and BGC (β-d-glucopyranose) to a blue circle. The anomeric form is mentioned explicitly for those cases where just one form is present in the PDB. As the SVG file format supports tooltips (messages that get displayed when the mouse hovers a graphical component), all information related to the linkages is displayed there in order to keep the diagrams minimal. This figure features all three-letter codes recognised by Glycoblocks up to the date of this publication.

Unlike proteins or nucleic acids, polysaccharides are frequently branched and in addition have two alternative configurations in their glycosidic linkages. While this imposes considerable restrictions on their three-dimensional conformations, it is precisely this nonlinear nature of glycans that poses a challenge in terms of two-dimensional representation. A number of sequence formats (*e.g.* LINUCS, Bohne-Lang *et al.*, 2001 ▸; GLYCO-CT, Herget *et al.*, 2008 ▸) have been developed for creating textual renditions of branched polysaccharides, each with their respective strengths and pitfalls. Many of the more complex formats are particularly well suited for conveying identification results from techniques such as mass spectrometry, and have been successfully used for mapping glycan sequences to proteoglycan structure entries in the Protein Data Bank (Berman *et al.*, 2003 ▸), effectively bridging the gap between two- and three-dimensional information (Campbell *et al.*, 2014 ▸). While most sequence formats provide machine-readable, univocal descriptions of glycan sequences, graphical conventions are better suited for human interaction and visualization. The graphical convention first introduced by Kornfeld *et al.* (1978 ▸) gained widespread popularity after being standardised (Varki *et al.*, 1999 ▸) and perfected (Varki *et al.*, 2009 ▸, 2015 ▸) to match the needs of the glycobiology community. This convention (hereafter termed the ‘Essentials’ notation) assigns a colour to the different stereochemistries occurring in glycans (*e.g.* blue, glucose; green, mannose; yellow, galactose; see Fig. 1 ▸) and identifies the different sugar types by altering each block’s shape (*e.g.* square, amino sugars; diamond, acidic sugars; or a white hexagon for unknown sugars; see Fig. 1 ▸), with α and β bonds being depicted as dashed and continuous lines respectively, a feature adopted from Oxford nomenclature (Harvey *et al.*, 2009 ▸). Also, connecting lines may be oriented according to the ring position where the linkage starts (*e.g.* a 45° rotation for a 1–6 link). Nowadays, it is even possible to employ the Essentials convention to search databases, such as UniCarbKB (Campbell *et al.*, 2014 ▸) or glycosciences.de (Loss & Lütteke, 2015 ▸), for particular glycans through the use of graphical tools such as *GlycanBuilder* (Damerell *et al.*, 2012 ▸).

With most of the biotechnological interest in glycosylation of proteins focused on how and where ligand carbohydrates appear in a protein–sugar complex structure, the important interactions provided by covalently attached glycans are often overlooked. These contacts have clear implications on correct glycoprotein folding and on protein–glycan and glycan–glycan recognition, for example having a clear impact on the thera­peutic effects of antibodies. Their importance has been evident for several years (Sinclair & Elliott, 2005 ▸), as they can show how and why the glycans are required. To some extent, it is possible to visualise them in two dimensions by using ligand-focused software such as *Ligplot+* (Laskowski & Swindells, 2011 ▸), or by accessing the online facilities provided by the PDB (Stierand & Rarey, 2010 ▸). Nevertheless, the number of contacts provided by glycosylation trees (which can be composed of more than ten monosaccharide units and may establish contacts with other equally complex glycans) limits this in practice to simple cases. As further a complication, glycans may also bind to aromatic residues (reviewed in Hudson *et al.*, 2015 ▸), which frequently line active sites in carbohydrate-active enzymes (Lombard *et al.*, 2014 ▸). Such well defined stacking interactions are visually evident but rarely identified in graphics programs, requiring scientists to follow complicated bespoke protocols for their depiction (*e.g.* creating two dummy atoms at the centre of each ring system and drawing a line between them).

Extracting visual information from densely populated scenarios requires simplification, *i.e.* not all information is relevant at the same time. Conceptualization in three dimensions has been successfully implemented already with the ribbon diagram (Richardson, 1985 ▸), which is the representation of choice for proteins, and has been implemented with minor local variations in all major structure visualization software. Perhaps unsurprisingly, carbohydrates have not been so fortunate in this regard and up until now only a handful of programs have introduced *ad-hoc* solutions that simplify sugar representation. For example, *SweetUnityMol* (Pérez, Tubiana *et al.*, 2015 ▸) converts monosaccharides into textured hexagonal shapes, which are then coloured based on an expansion of the Essentials colour scheme, while the *Azahar* plugin for *PyMOL* (Schrodinger, 2015 ▸) also produces hexagonal shapes that can be coloured based on a set of predefined choices. However, to the best of our knowledge none of these programs are able to produce shapes that match those specified in the standard Essentials notation, nor have they extended functionality for simplifying complex interactions.

Here, we introduce a schematic three-dimensional representation that minimises graphic complexity while retaining the visual identification, spatial orientation and branching structure of the monosaccharides. This feature, named Glycoblocks, is available as part of *CCP4mg*, the *CCP4 Molecular Graphics program* (McNicholas *et al.*, 2011 ▸), which is distributed as part of the *CCP*4 suite (Winn *et al.*, 2011 ▸). *CCP4mg*, incorporating *Privateer* (Agirre, Iglesias-Fernández *et al.*, 2015 ▸), a required component, is alternatively available as a standalone program (http://www.ccp4.ac.uk/MG). Glycoblocks aims to provide a simplified view for glycans, similar to what the ribbon diagram achieved for proteins, reducing each entity and interaction to an easily identifiable three-dimensional sketch.

## 2. Materials and methods   

### 2.1. Graphic conventions   

An analysis of N- and O-glycans in the PDB (Agirre, Davies *et al.*, 2015 ▸) performed with the *CCP*4 program *Privateer* (Agirre, Iglesias-Fernández *et al.*, 2015 ▸) identified which sugars were present in the deposited structures, generating a list of three-letter codes from the PDB’s chemical component dictionary. *Privateer*’s most recent version (MKIII) introduced a Python scripting interface for seamless integration into other programs and pipelines (*e.g.* *CCP4mg* and *CCP4i2*, which handle most of their logic in Python code). The functions in this interface produce validation data in extensible markup language (XML) format, with scalable vector graphics (SVG) two-dimensional diagrams of the glycan structures being embedded in the XML output. These diagrams are encoded according to the most recent Essentials notation (Varki *et al.*, 2015 ▸), with dashed lines for α-bonds and continuous lines for β-bonds, a feature that the Essentials system has recently adopted from the Oxford nomenclature (Harvey *et al.*, 2009 ▸). For added interactivity, they are annotated with all the validation information produced by *Privateer* (checks on stereo- and regiochemistry, ring puckering and conformation, and linkage torsions, all available as a tooltip), and with HTML links containing MMDB (the CCP4 coordinate library; Krissinel *et al.*, 2004 ▸) residue selections, which *CCP4mg* is able to process in order to focus on the selected sugar upon clicking on a two-dimensional shape (sugar, amino acid or link). These diagrams can be shown by choosing *Glycan viewer* from the *CCP4mg* menu.

**Figure 2:**
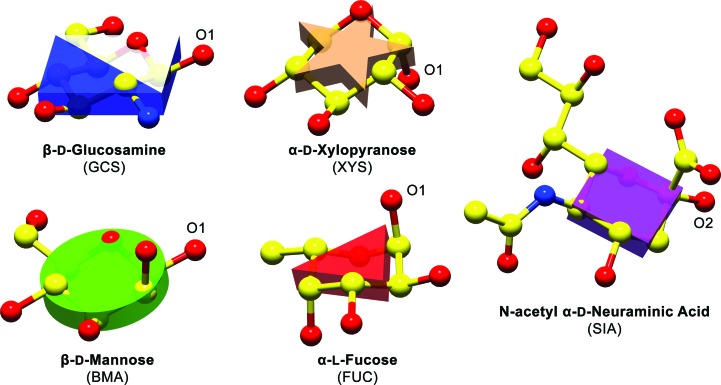
Orientation of Glycoblocks with respect to the atomic models they represent. All monosaccharides have been oriented with the oxygen linked to the anomeric carbon (see annotations on the picture) on the right. Despite the d- and l-sugars showing ^4^C_1_ and ^1^C_4_ conformations, respectively, the orientation of the block remains representative, providing a clear hint at the stereochemistry. For clarity, object outlines and H atoms have been omitted.

In Glycoblocks the Essentials notation (Varki *et al.*, 2009 ▸, 2015 ▸) has been translated into three-dimensional solids, matching each shape, sugar name and colour with the corresponding three-letter codes recognised by *Privateer* (Fig. 1 ▸). Each Glycoblock is a vertical extension of the original two-dimensional shape, producing a triangular prism for fucose, a rectangular prism for *N*-acetylgalactosamine or a cylinder for mannose (Fig. 2 ▸). In order to provide a notion of the particular orientation of the sugars, the Glycoblocks are oriented according to the mean ring plane, defined in the following section.

As *Privateer* is able to detect covalently linked N-, O- and S-glycans, *CCP4mg* shows them automatically in the Glycoblocks representation upon loading the structure of a glycoprotein. Although less frequently used, the representation may also be selected when examining ligand mono- and polysaccharides, provided that they are identified using monosaccharide three-letter codes, *i.e.* two β1,4-linked glucoses (BGC) and not a single cellobiose entity (CBI). This requirement is not expected to have a negative impact, as the majority of sugar structures have been deposited using the monosaccharide codes. Nevertheless, common di- and oligosaccharides will be added to the Glycoblocks representation in a forthcoming update. While the Essentials notation covers most of the N- and O-glycan-forming carbohydrates, unknown sugars can be depicted as white/grey hexagons (Fig. 1 ▸), with the first letter of the three-letter code being shown in the two-dimensional diagram for quick identification. In *CCP4mg*, these are shown as grey hexagonal prisms.

### 2.2. Computing interactions   

**Figure 3:**
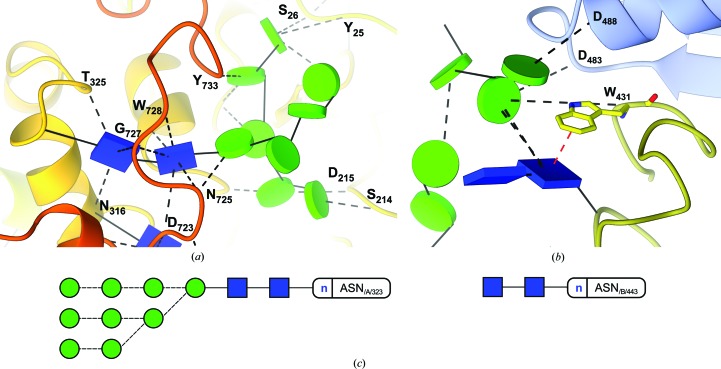
Visualizing interactions with Glycoblocks. In the figure, the structure of a heavily glycosylated fungal glycosylhydrolase (PDB code 5fjj), reported by Agirre *et al.* (2016 ▸). (*a*) View of the interactions of a high-mannose tree. The glycan connected to Asn323 is perhaps the only example of a three-dimensional structure of a complete high-mannose tree in the PDB. As the protein part has been coloured in rainbow style, it can immediately be seen that the glycan establishes hydrogen bonds across multiple domains and with other glycans which, in turn, interact with other parts of the protein. (*b*) Visualizing stacking interactions. The first GlcNAc sugar is linked in a flipped conformation to Asn443 due to the stacking interaction with Trp431 (W_431_ in the picture). These interactions are depicted in red. (*c*) Two-dimensional representation by *Privateer*. Dashed lines indicate an alpha link.

**Figure 4:**
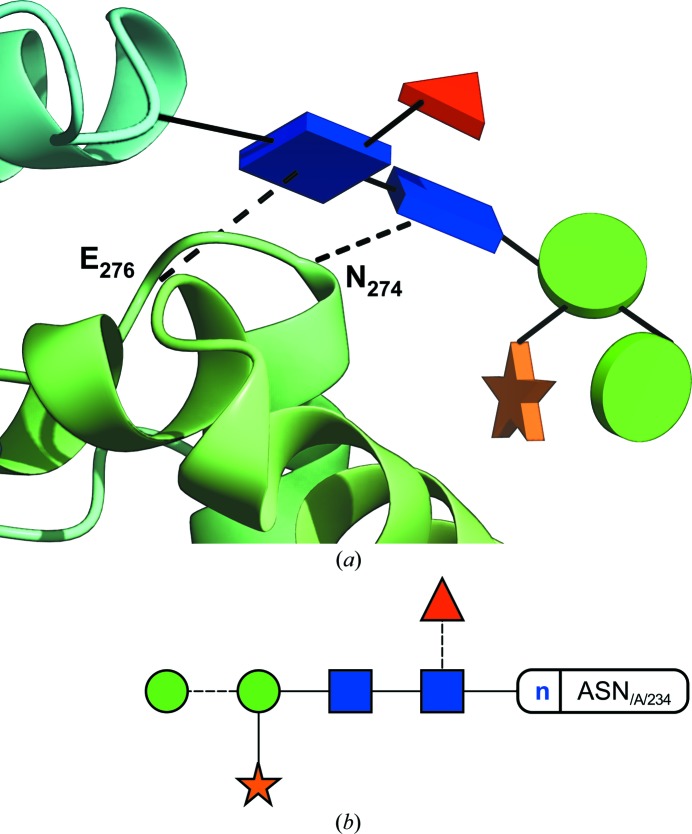
(*a*) Glycoblocks representation of a plant N-glycan and its interactions (PDB code 5aog). The structure depicted is a cationic class III peroxidase purified from *Sorghum bicolor* (Nnamchi *et al.*, 2016 ▸), which shows the typical core α1,3-fucosylated glycans covalently attached to it. The two core GlcNAc sugars establish two hydrogen bonds (dashed lines in the three-dimensional view), respectively, to one end of a neighbouring α-helix. (*b*) Two-dimensional representation produced by *Privateer*. Dashed lines indicate an α-link.

**Figure 5:**
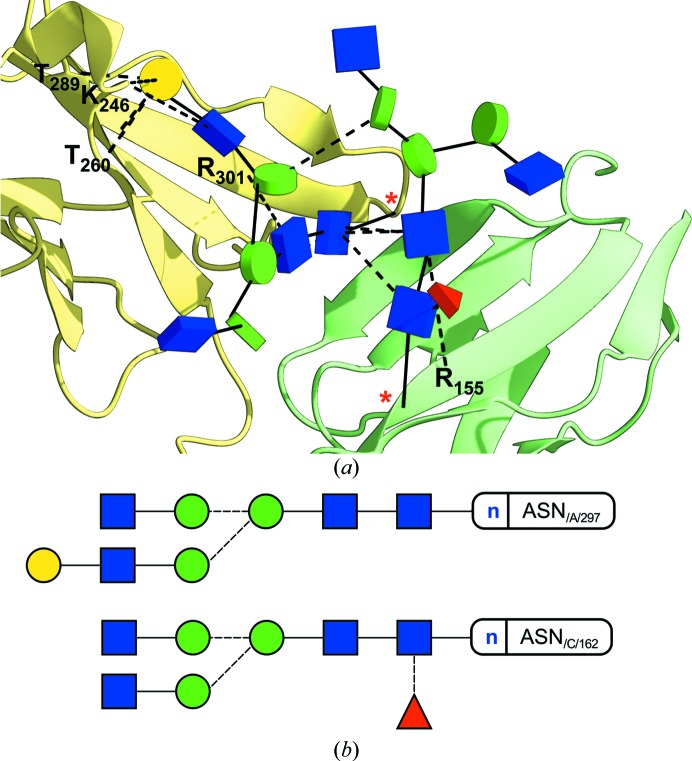
Glycan–glycan and glycan–protein contacts in an antibody–Fc γ receptor IIIa (FcγRIIIa) complex (PDB code 4a5t). (*a*) Glycoblocks representation. The non-fucosylated Fc fragment has been coloured in yellow, FcγRIIIa is in green. Most of the contacts that bind the two structures together occur between the glycans themselves. The missing fucose residue would have appeared at the interface between both chains, causing steric hindrance according to the authors (Mizushima *et al.*, 2011 ▸). The glycosylation points (asparagine residues 297 and 162) have been marked with a red asterisk. (*b*) Two-dimensional representation produced by *Privateer*. Dashed lines indicate an α-link.

**Figure 6:**
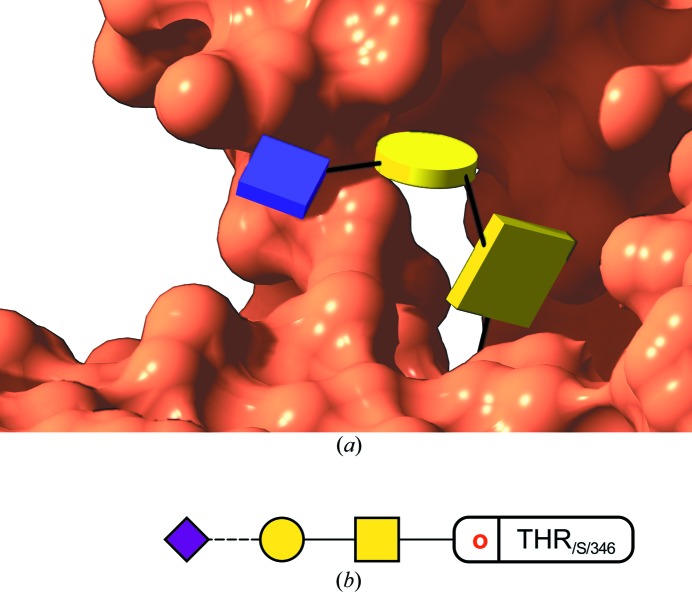
(*a*) View of an O-glycan. This shows one of the rare examples of O-glycosylation found in the PDB (code 4a5t, solved to 3.49 Å resolution), reported by Xue *et al.* (2012 ▸). As can be seen from the two-dimensional diagram (see *b*), the GalNAc–Thr linkage was originally modelled as β whilst in reality it had to be α. It is only by using all available knowledge of glycochemistry that these mistakes can be avoided, as the fit to a featureless map must always be tightly restrained to what is known in terms of link distances, angles and torsions. (*b*) Two-dimensional representation by *Privateer*. The dashed line indicates an α-link.

**Figure 7:**
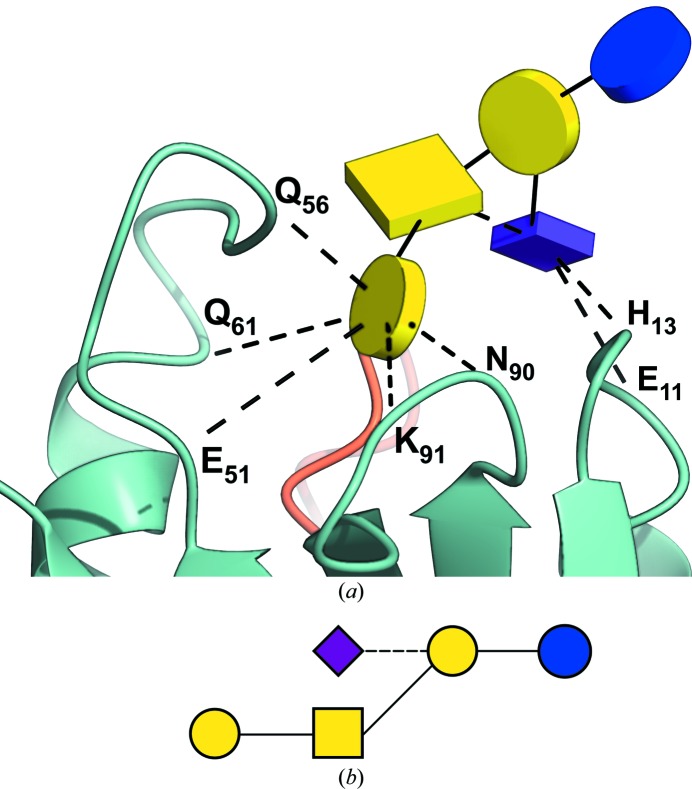
Visualizing ligand glycans. (*a*) A simplified three-dimensional view of the interactions between the GM1 pentasaccharide and the subunit B5 of the choleratoxin pentamer (PDB code 3chb), reported in Merritt *et al.* (1994 ▸) and re-refined in Merritt *et al.* (1998 ▸). Only direct hydrogen bonds are shown, as waters have been omitted from the picture. The protein part has been coloured by chain. There is an unlabelled hydrogen bond between the GalNAc and Neu5Ac monosaccharides, also drawn as a dashed line. All the depicted interactions, computed on the fly by *CCP4mg*, match those manually determined in the original research (Merritt *et al.*, 1994 ▸). (*b*) Two-dimensional representation by *Privateer*. The dashed line indicates an α-link.

Hydrogen bonds are depicted using a dashed line from each block’s centre to the C_α_ of the amino acid with which it interacts in the protein backbone (Figs. 3 to 7), the hydrogen bonds being computed internally by *CCP4mg* (Potterton *et al.*, 2002 ▸, 2004 ▸; McNicholas *et al.*, 2011 ▸). Covalent bonds, including the protein–glycan ones such as Asn^ND2^–GlcNAc^C1^, are depicted as solid lines, with each linkage arising from the projected side of the glycoblock. Stacking interactions are computed according to the criterion defined by Hudson *et al.* (2015 ▸), whereby interaction distances must fall within a 4 Å limit and the angle formed by vectors orthogonal to the aromatic and mean carbohydrate planes must not exceed 30°. These are depicted as red dashed lines between each ring’s centre of mass.

Linkages are determined by chemistry perception instead of relying on the deposited LINK records, as it has been reported that many structures contain wrongly specified links (Lütteke *et al.*, 2004 ▸; Lütteke & Lieth, 2009 ▸). Distances in Å, corresponding to the actual distance between the two atoms forming the bond, and residue numbers can be optionally annotated adjacent to each line, providing quantitative details on the interactions. Thickness and size may be changed for bond cartoons and blocks, with the default values having been optimized for close-up views. For reasons of clarity, the bonding network is not shown by default and has to be activated from the *Preferences* menu.

### 2.3. Orientation of the blocks   

Let *i, j* and *k* represent three consecutive atoms in a sugar ring of size *s* and 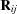 the position vector that goes from atom *i* to atom *j*, identifying the bond between both atoms. A vector 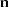 is then calculated 
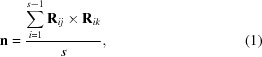
with 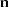 normal to the plane that will define the block’s orientation.

### 2.4. Figure preparation   

All three-dimensional figures have been produced with *CCP4mg* (McNicholas *et al.*, 2011 ▸) and all two-dimensional vector diagrams with *Privateer* (Agirre, Iglesias-Fernández *et al.*, 2015 ▸), as *CCP4mg* shows interactive versions of these on-screen, but does not offer an option to save them to disk. The *Shadows, Occlusion* and *Object outlines* options were activated under the *Lighting* menu in *CCP4mg*, and labels were added using the same program. Interactions were represented as dashed cylinders.

## 3. Discussion   

The Glycoblocks representation was originally developed to simplify the visualization of the complex interactions that can be found in heavily glycosylated structures, such as those in Agirre *et al.* (2016 ▸). Reducing a 12-atom-plus entity to a single block while retaining its overall orientation and link cardinality greatly helps uncluttering the view of a glycan. In addition, depicting covalent, stacking and close-range electrostatic interactions as lines between the blocks and the C_α_ from the linked residue, allows for the removal of the side chains from complex interaction scenarios. The integration of Glycoblocks into *CCP4mg* makes it instantly possible to represent interactions between monomers related by crystallographic symmetry, to create movies that can be integrated in slide shows, or to generate stereoviews with an enhanced sense of depth (Agirre *et al.*, 2016 ▸).

The representation has been tested in most practical scenarios with positive results, summarised as follows.

### 3.1. High-mannose N-glycans   

Of simple composition (9 × Man and 2 × GlcNAc, arranged in up to three branches), high-mannose glycans can establish hydrogen bonds with other domains or even chains of the protein to which they are attached, as distant from the original glycosylation point as 30 Å (Fig. 3*a* ▸). These glycans are typically seen as structural reinforcement for glycoproteins, with mostly intact trees being found linked to asparagine residues in the core of the protein, and shorter trees or even single GlcNAc monosaccharides (possibly a result of the action of endoglycosidases) appearing more frequently towards the surface (Agirre *et al.*, 2016 ▸). While they are far more frequent in binding sites (Hudson *et al.*, 2015 ▸), stacking interactions may play a role on the conformation of glycans too; particular linkage conformations can be enforced when aromatic residues are in the neighbourhood (Fig. 3*b* ▸). These are depicted in the Glycoblocks representation as a red dashed line, which can be traced to the centre of mass of the aromatic residue or to the C_α_, similar to how hydrogen bonds are shown.

### 3.2. Plant glycans   

Common plant complex N-glycans include core α1,3-fucose (not to be confused with the core α1,6 linkage found in mammalians) and β1,2-linked xylose saccharides (Fig. 4 ▸), which are added during the final stages of processing in the Golgi apparatus (Strasser, 2014 ▸). Their function remains uncertain, although their tendency to show up on the protein’s surface hints at their potential implications in recognition processes.

### 3.3. Antibodies   

N-glycosylation is a key functional part of antibodies, vital for their structure and interactions and hence for their thera­peutic effectiveness. In Fig. 5 ▸, a glyco-engineered Fc fragment lacking core-linked fucose binds to the human Fc γ receptor IIIa (FcγRIIIa) through a network of hydrogen bonds (Mizushima *et al.*, 2011 ▸). This non-fucosylated variant was shown to have stronger affinity to FcγRIIIa because of reduced steric hindrance in the region where the core α1,6-fucose would be found in the fucosylated form. A second example using antibodies (PDB code 4byh; Crispin *et al.*, 2013 ▸) can be found in the supplementary video protocol.

### 3.4. O-GalNAc glycans   

O-glycosylation, only known to be present in eukaryotes, groups the covalent modification of a serine or threonine residue with, most frequently, *N-*acetylgalactosamine (GalNAc, yellow square in Fig. 1 ▸), although other modifications do exist, such as O-linked mannose, fucose, xylose, galactose, glucose or, notably, the intracellular *O*-GlcNAc modification with *N-*acetylglucosamine. *O*-GalNAc glycans, usually found linked to mucins, have implications in many signalling and communication processes occurring for instance in cancer, including metastasis formation (Pinho & Reis, 2015 ▸). In addition to over- or under-expression, structural changes in *O*-GalNAc glycans can be associated with certain types of cancer and, therefore, be used as biomarkers for diagnosis (Tuccillo *et al.*, 2014 ▸). A partial *O*-GalNAc glycan can be seen attached to human native plasminogen (PDB code 4a5t) in Fig. 6 ▸, with sialic acid (Neu5Ac) at its terminus. This structure was determined at low resolution (3.49 Å), and contains modelling errors such as a wrong GalNAc–Thr linkage, which must be α. These problems become apparent in the resulting two-dimensional diagrams, which incorporate anomeric information on the linkages (dashed *versus* continuous line).

### 3.5. Ligand glycans   

The GM1/choleratoxin B-pentamer complex (Merritt *et al.*, 1994 ▸, 1998 ▸) is a classic example of an intricate bonding network between a ligand glycan and a protein. Despite having waters removed from their original three-dimensional stereographic depiction, the interaction network proved visually challenging to interpret, and had to be explained in an expanded, cleverly drawn planar diagram (Merritt *et al.*, 1994 ▸). In Fig. 7 ▸, a Glycoblocks three-dimensional interpretation of this scenario provides a simplified way of looking at the same interactions, reducing the number of atoms and dashed lines to a minimum and eliminating the need for a stereo figure.

### 3.6. Analysing NMR structures   

**Figure 8:**
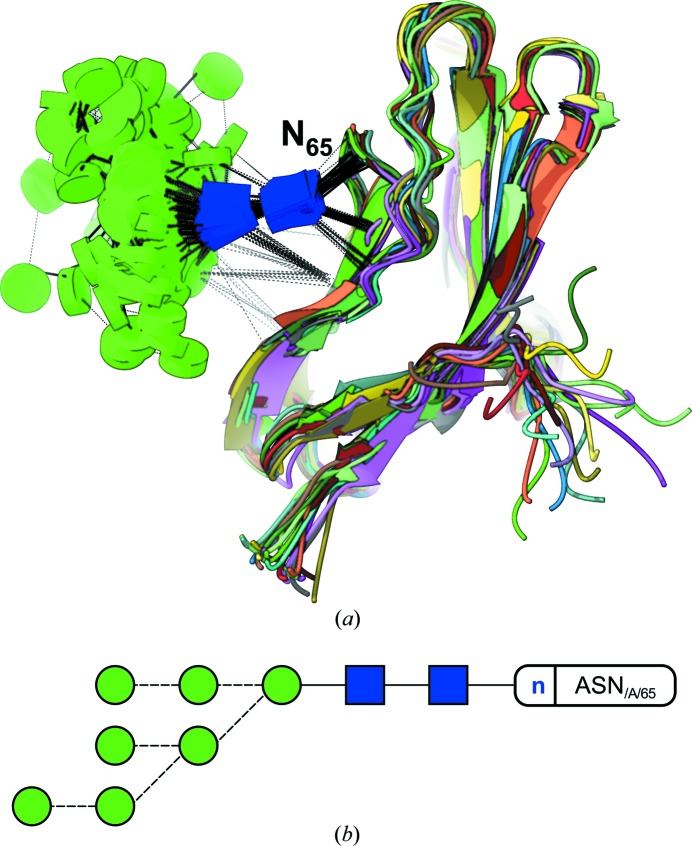
Simplifying NMR model representation. (*a*) Glycoblocks view of a partial high-mannose glycan N-linked to the adhesion domain of human CD2. This lateral view of the glycoprotein allows for an unobscured way of looking at the contacts that occur between sugars, and sugars and protein. While hydrogen bonds keep the two core GlcNAc sugars tied to the protein, the rest of the glycan shows great conformational variability. The protein part has been coloured by model. (*b*) Two-dimensional representation by *Privateer*. Dashed lines indicate an α-link.

Removing glycans from the surface of a glycoprotein enzymatically (*e.g.* using EndoH) has become standard practice in X-ray crystallography whenever the first crystallization trials fail. Other techniques, such as NMR, are able to cope with the glycans’ conformational variability and thus represent a suitable alternative for those cases when the external glycans are not an obstacle but the very target of the study, *e.g.* in those cases where terminal sugars play a central role in molecular recognition (Ardá *et al.*, 2013 ▸; Canales *et al.*, 2013 ▸). An example is shown in Fig. 8 ▸, where the glycan takes part in counterbalancing the positive charge density near the glycosylation point (N_65_ in the figure) in the adhesion domain of human CD2 (Wyss *et al.*, 1995 ▸).

### 3.7. Block orientation and stereochemistry   

A decision was made neither to use regular polyhedra nor spheres; instead prisms or cylinders are cut thin by two parallel planes (bases) which are orthogonal to the sides. This has two benefits: the sugar’s orientation can be retained in the block representation; and these occupy a similar volume on-screen. The different orientation between two blocks can hint at their linkage’s torsions, and that way unusual linkage conformations can be ascertained from the pictures (see Fig. 3*b* ▸).

## 4. Conclusions   

The possibility of detecting glycans in structures will enable databases such as the PDB (Berman *et al.*, 2003 ▸) or Glyco3D (Pérez, Sarkar *et al.*, 2015 ▸) to display images in Glycoblocks format whenever glycans are found in a structure. Embedding validation information in non-intrusive tooltips should encourage users to adopt a critical view on the sometimes subjective and debatable interpretations that can be found in the PDB (Lütteke *et al.*, 2004 ▸; Crispin *et al.*, 2007 ▸; Lütteke & Lieth, 2009 ▸; Agirre, Davies *et al.*, 2015 ▸).

## Supplementary Material

Video protocol. DOI: 10.1107/S2059798316013553/ba5253sup1.mp4
